# Carriage of antibiotic-resistant *Enterobacteriaceae* in hospitalised children in tertiary hospitals in Harare, Zimbabwe

**DOI:** 10.1186/s13756-016-0155-y

**Published:** 2017-01-11

**Authors:** Marcelyn T. Magwenzi, Muchaneta Gudza-Mugabe, Hilda A. Mujuru, Mutsa Dangarembizi-Bwakura, Valerie Robertson, Alexander M. Aiken

**Affiliations:** 1 College of Health Sciences, Department of Medical Microbiology, University of Zimbabwe, PO Box A178, Harare, Zimbabwe; 2 National Microbiology Reference Laboratory, Harare Central Hospital, Harare, Zimbabwe; 3 College of Health Sciences, Department of Paediatrics and Child Health, University of Zimbabwe, Harare, Zimbabwe; 4 Harare Central Hospital, Harare, Zimbabwe; 5 Parirenyatwa Group of Hospitals, Harare, Zimbabwe; 6 Biomedical Research and Training Institute, Harare, Zimbabwe; 7 London School of Hygiene and Tropical Medicine, London, UK

**Keywords:** *Enterobacteriaceae*, ESBL, Antibiotic resistance, Colonization, Children

## Abstract

**Background:**

Extended-spectrum β-lactamase-producing and gentamicin resistant *Enterobacteriaceae* are increasingly recognised as a major cause of infection in low-income countries. We assessed the prevalence of gastrointestinal carriage of these bacteria in hospitalised children in Harare, Zimbabwe.

**Methods:**

We conducted a cohort study in paediatric inpatients at two tertiary-referral hospitals between May and July 2015. Rectal swabs and faecal samples were collected within 24 h of admission and further follow-up samples were collected on alternate days during hospitalization. Disc-based, selective and enrichment methods were used to detect carriage of these two forms of resistance. Standard methods were used to confirm resistance status and determine the susceptibility of resistant isolates to other commonly-used antibiotics.

**Results:**

One hundred and sixty four paediatric inpatient admissions (median age = 1.0 year, IQR = 0.2–2.2years) were enrolled, and an average of 1.9 faecal samples per patient were collected. On admission, 68/164 (41%) patients had both ESBL and gentamicin-resistant *Enterobacteriaceae* detected, 18 (11%) had ESBL only, 17 (10%) had gentamicin resistance only and 61 (37%) had negative screening for both forms of resistance. During hospitalisation, 32/164 (20%) patients were found to have a type of resistant organism which was not present in their admission sample. We found that faecal samples and use of a selective enrichment broth enhanced the detection of resistant organisms. Amongst resistant bacteria isolated, there were high levels of resistance to ciprofloxacin and chloramphenicol, but not ertapenem.

**Conclusions:**

More than half of children had enteric carriage of a clinically-relevant form of antibiotic resistance on admission to public-sector hospitals in urban Zimbabwe. Additionally, a fifth of children acquired a further form of resistance during hospitalisation. Urgent action is needed to tackle the spread of antibiotic resistant enteric bacteria in African hospitals.

## Background

Clinically significant levels of resistance to various antibiotic classes have developed in most Gram-negative bacteria, though there is marked geographic variation in the prevalence of resistance [1]. Historically, many forms of antibiotic resistant organisms emerged in and were disseminated from healthcare facilities in high-income settings, but in more recent times, hospitals in low and middle-income settings appear to have been the origin and amplifiers of several forms of resistance [2–4]. Weak antibiotic stewardship and limited infection control resources promote the spread of antibiotic resistant organisms in hospitals, especially where there are no microbiological diagnostic services for the identification of resistance [5].

In the family of *Enterobacteriaceae,* organisms such as *Escherichia coli* and *Klebsiella pneumoniae* can produce extended-spectrum β-lactamase (ESBL) enzymes, which confer resistance to third generation cephalosporins and most penicillins but are inhibited by agents such as clavulanate. These types of resistant bacteria are increasingly implicated as causes of both community- and hospital-acquired infections [6, 7] and are associated with higher levels of mortality than non-ESBL producers [8]. Across sub-Saharan Africa, a limited number of studies have described high levels of ESBL faecal carriage in children in association with hospital facilities [9–13]. Gentamicin is an aminoglycoside antibiotic that has been used for the treatment of Gram-negative infections for more than 40 years [14]. Its low-cost and broad spectrum of activity means that it is widely used for treatment of severe infections in many low-income countries.

The gastrointestinal tract is the principal ecological niche for *Enterobacteriaceae*. In this environment, inter- and intra-species exchange of resistance genes can occur and under appropriate selective pressure, resistant species can rapidly emerge and dominate [15]. While faecal carriage of antibiotic resistant bacteria is not an immediate threat to a healthy individual, it poses two risks—firstly, when auto-infection of a sterile body-site subsequently occurs (for example, hospital-acquired bacteraemia [16]) treatment is substantially more difficult and secondly, there may be transmission of the resistant organism to other individuals [17].

In order to describe levels of carriage of Gram-negative antibiotic resistance amongst hospitalised children in Zimbabwe, we aimed to measure 1) the prevalence of carriage of ESBL producing and gentamicin-resistant enteric bacteria in paediatric patients at the time of admission to hospital and 2) the risk of acquisition for the same types of bacteria during the corresponding time of hospitalization. We also sought to evaluate different methods for screening for the presence of these resistant organisms.

## Methods

### Study design

This was a prospective observational cohort study conducted in paediatric wards at two tertiary government hospitals in Harare, Zimbabwe between May and July 2015. Paediatric patients were recruited within 24 h of admission to hospital and remained in the study until discharge from hospital or death. All paediatric patients were eligible for inclusion in the study regardless of their presenting illness, provided that they remained in hospital for at least one night. These hospitals had paediatric wards divided into cubicles of 6 to 8 beds and limited isolation rooms. Each cubicle or room had a single hand-wash basin; alcohol-based hand sanitizers were not consistently available. There was no established practice of screening for carriage of resistant organisms in these wards at the time of this study.

### Screening for ESBL and gentamicin resistance

Two rectal swabs and/or a stool sample were collected within 24 h of admission. Further similar samples were collected three times per week for all participants. The swabs and stool samples were directly inoculated onto a range of screening plates in the Department of Medical Microbiology laboratory, University of Zimbabwe, as follows:

1. MacConkey agar without salt (Oxoid, UK) plates with either a Cefpodoxime (10 μg) or a Gentamicin (10 μg) disc placed on the plate. The plates were incubated at 37 °C for 24 h. Growth within 5mm of the margin of the disc was considered potentially significant.
2. Selective ChromID ESBL-agar (Biomerieux, France) and Gentamicin-MacConkey plates. The latter was prepared in-house by adding 800 μl Gentamicin 10 mg/ml (Gibco, UK) in 1 L of MacConkey agar without salt. Any growth on these plates was considered potentially significant.
3. Samples were selectively enriched overnight in 5 ml nutrient broth with either a Cefpodoxime (10 μg) or a Gentamicin (10 μg) disc added to the bijou bottle. These enriched samples were sub-cultured the following day and then handled in the same way as the direct methods above.

Where results were discordant between different sample types from the same patient using the screening methods, any detection of a resistant organism was considered to be the correct identification. A single colony of each distinguishable type (up to three per sample) was picked from a resistance screening plate, grown on a purity plate and subsequently tested as described below.

### Identification of isolates

Bacterial isolates expressing either ESBL or Gentamicin resistance or both were obtained. An oxidase test was performed (Remel Bactidrop, UK) for all isolates. Oxidase-negative isolates were identified to the family/genus level using API10S kits (Biomerieux, France) according to the manufacturer’s instructions; this system does not consistently provide identification to the species level. The ESBL phenotype was confirmed by paired disc tests [18] according to the manufacturer’s instructions (Mast Diagnostics, UK). Where a sample had no isolates confirmed as ESBL producers by paired disc testing, we considered the sample to be negative for that type of resistance throughout the analysis.

### Antimicrobial susceptibility testing

Overnight pure cultures confirmed to be ESBL positive or gentamicin resistant had further susceptibility testing performed using the standard disc diffusion method based on Clinical Laboratory Standards Institute (CLSI) breakpoints for susceptibility testing. The antibiotics tested were gentamicin (10 μg), chloramphenicol (30 μg), ciprofloxacin (1 μg), ertapenem (10 μg), cefpodoxime (10 μg) and ampicillin (10 μg) (Oxoid, England). Reference strains *E. coli* ATCC25922 (fully susceptible) and *E. coli* NCTC13353 (ESBL CTX-M type and gentamicin resistant) were used as control organisms. Data were recorded in a Microsoft Access database and STATA v13 was used to calculate prevalence and acquisition rates of resistance carriage and perform tests of association.

## Results

### Admissions

A total of 164 paediatric inpatients were recruited to the study out of a total of 1757 paediatric admissions to these two hospitals in the study period. Most patients (142/164, 87%) were recruited on the day following their admission to hospital (hence after spending one night in hospital), but all within 24 h of their admission. A summary of these patients is shown in Table 1. The median age was 1.0 years (IQR 0.2 to 2.2 years). The most commonly recorded admission diagnoses were “severe pneumonia”, “sepsis” and “severe malnutrition”. The average duration of hospitalization was 5.6 days (IQR 2–7.5 days; range 1–32 days) and the inpatient mortality was 8% (13 deaths/164 admissions).

**Table 1:** Summary of study admissions

	Number (*n* =164)	%
Age of child		
0–28 days	19	12%
29 days–1 year	63	38%
1–5 years	64	39%
Over 5 years	18	11%
Ward of recruitment		
Harare Childrens Hospital, ward B1	41	25%
Harare Childrens Hospital, ward B2	63	38%
Parirenyatwa Hospital, admission unit	3	3%
Parirenyatwa Hospital, ward A4 General	22	22%
Parirenyatwa Hospital, ward A4 Special	24	24%
Parirenyatwa Hospital, ward A5	11	11%
Enteric carriage of resistance on admission		
Both ESBL and gentamicin resistance	68	41%
ESBL resistance only	18	11%
Gentamicin resistance only	17	10%
Neither form of resistance	61	37%
Outcome of admission		
Discharged	151	92%
Died	13	8%

### Carriage of antibiotic resistance on admission

The prevalence of ESBL carriage was 52% (86/164) and gentamicin resistance carriage was 52% (85/164), on the initial sample taken from patients, with 41% of all patients having both forms of resistance. On admission, ESBL carriage was higher amongst children in the age groups 29 days-1 year (39/63; 62%) and 1–5 years (37/64; 58%) and lower in the younger (0–28days (7/19; 37%)) and older (5+ years (3/18; 18%) age groups; (Fishers exact test across these categories *p* = 0.001). We found no difference in carriage rates by age group for gentamicin resistance on admission to hospital. Only 37% of children had neither form of resistance detectable—see Table 1. There was no association between hospital of admission and prevalence of carriage of ESBL (χ^2^-test *p* = 0.23) or gentamicin resistance (*p* = 0.32). Death as an inpatient was associated with ESBL carriage on admission (Fisher’s exact test *p* = 0.02) but not with gentamicin resistance (*p* = 0.25).

### Acquisition of carriage during admission

**Fig. 1:**
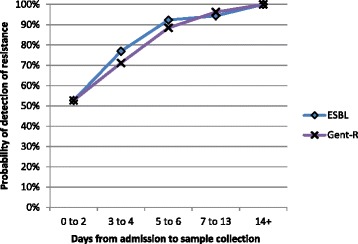
Probability of detection v time-to-sample

An average of 1.9 samples were obtained per patient (range = 1 to 8) with 91 patients having two or more samples collected. A total of 32 admissions were detected to have a new form of resistance during their hospital admission: 17 gained both ESBL and gentamicin-resistance, 12 gained ESBL only and 3 gained gentamicin resistance only (see Table 2). There was no association between hospital and acquisition of resistance during admission for either ESBL (χ^2^-test *p* = 0.40) or gentamicin resistance (χ^2^-test *p* = 0.40). The probability of any sample containing a resistant organism was closely related to the time since admission to hospital—see Fig. 1. After a patient had been hospitalised for five or more days, there was over 90% probability (86/93 samples) that carriage of both ESBL and gentamicin-resistant organisms would be detected in a faecal specimen.

**Table 2:** Summary of ESBL or Gentamicin resistance acquisition status during admissions

>1 faecal sample taken during hospital admission	91/164 (55%)
New detection of ESBL during admission	29/164 (18%)
New detection of gentamicin resistance during admission	20/164 (12%)
New detection of either form of resistance	32/164 (20%)
New detection of both forms of resistance	17/164 (10%)

### Screening methods for detection of resistant organisms

A total of 312 samples were collected, of these 143 were rectal swabs only, 23 were faecal samples only and 146 had both types of specimen. In the samples with both rectal swabs and faecal samples, the sensitivity of the different detection techniques is shown in Table 3. Broadly speaking, we found that higher sensitivity was achieved with faecal samples and inclusion of the enrichment broth step. Use of selective media did not achieve substantially higher sensitivity, but was noted to be practically easier to use in the laboratory. Out of the oxidase-negative Gram-negative isolates detected by the ESBL screening methods and confirmed as *Enterobacteriaceae*, the large majority (190/214; 89%) were confirmed as ESBL by both disc pairs (cefotaxime/clavulanate pair and ceftazidime/clavulanate pair) and some were confirmed by only a single pair of ceftazidime/clavulanate (16/214; 7.5%). A small number of isolates were not confirmed as ESBL-producers by either disc pair (8/214; 4%).

**Table 3:** Sensitivity of screening methods for resistant organisms (*n* = 146 samples)

	ESBL		Gentamicin-resistant	
Sample type	Rectal swab	Faecal sample	Rectal swab	Faecal sample
Plate with disc	73%	84%	66%	83%
Selective plate	75%	87%	70%	82%
Enrichment broth THEN plate with disc	90%	96%	87%	95%
Enrichment broth THEN selective plate	90%	96%	84%	96%

### Antibiotic resistance profiles

Amongst the isolated *Enterobacteriaceae* that were confirmed ESBL (*n* = 206) or gentamicin resistant (*n* = 146), we examined the co-resistance to other commonly used antibiotics—see Tables 4 and 5. We found high levels of co-resistance to ampicillin, ciprofloxacin and chloramphenicol, but virtually no co-resistance to ertapenem.

**Table 4:** Susceptibility profiles of confirmed ESBL-producing isolates (*n* = 206)

Antibiotic	Number resistant	% Resistant	Total tested
Gentamicin	130	65	199
Ampicillin	199	100	199
Ciprofloxacin	162	82	198
Chloramphenicol	105	53	198
Ertapenem	2	1	199

**Table 5:** Susceptibility profiles of Gentamicin resistant isolates (*n* = 146)

Antibiotic	Number resistant	% Resistant	Total tested
Cefpodoxime^a^	126	86	146
Ampicillin	144	99	146
Ciprofloxacin	112	77	146
Chloramphenicol	77	53	146
Ertapenem	0	0	145

^a^These isolates were not formally confirmed as ESBL-producers by double disc testing

## Discussion

Our study investigated enteric carriage of ESBL-producing and gentamicin resistant enteric bacteria in a sample of 164 hospitalised children drawn from two tertiary public-sector hospitals in Harare, Zimbabwe over a 3 month period. The main findings were high rates of carriage of ESBL and gentamicin resistant bacteria on admission to hospital and high rates of acquisition for the same resistance types during the inpatient period.

An initial sample was collected within 24 h of hospital admission for each patient, so these samples are likely to represent the carriage status prior to the current hospital admission. Other studies of ESBL prevalence around the time of admission to hospital have found carriage rates of 33% (133/408) in Guinea Bissau [9], 34% (37/110) in Gabon [12] and 22% (54/244) in Madagascar [11]. Amongst neonates in Tanzania, 25% (32/126) were found to have ESBL carriage, predominantly *Klebsiella pneumoniae* [13]. In a study conducted in a renutrition centre in Niger [10], ESBL carriage was 31% (17/55 patients) on admission and by time of discharge a further 15/16 children had acquired carriage. Our study, showed a higher prevalence of carriage than those previously described levels, though our methods of screening were probably more sensitive than the direct plating of rectal swabs used elsewhere. We are not aware of any previous African studies specifically investigating carriage of gentamicin resistance in hospitalised children. Carriage of resistance amongst hospital admissions is liable to be higher than carriage in the general community, as sick individuals probably have exposure to various healthcare-related risk factors prior to admission, so we cannot compare these results to truly community-based studies. However, the extremely high levels of carriage rates found suggest that circulation of ESBL and gentamicin resistance probably occurs in the community in Harare. Although we found an association between ESBL carriage and inpatient death, we were not able to assess for the confounding effects and hence it remains uncertain whether this is a causal association.

Collection of multiple follow-up samples during the inpatient period allowed us to observe acquisition of carriage of resistance over time. An important question is whether these in-hospital acquisitions are occurring via endogenous (arising from undetectably low levels already present in the gastrointestinal tract) or exogenous (arising from cross-infection from an external source) routes. This distinction has important implications for antibiotic stewardship and infection control measures. For endogenous routes of acquisition, antibiotic stewardship activities focussing on reducing the selective pressure from antibiotic prescribing are important. For exogenous acquisition, measures to minimise cross-infection, such as improved hand hygiene, are priorities. Whilst we cannot be certain, we feel that both routes are likely to be contributing to the high levels of nosocomial acquisition seen here. Our screening methods included an enrichment step so that we should have been able to detect small quantities of resistant bacteria, making endogenous acquisition less likely, though a single faecal specimen is not a representative sample of the estimated 10^14^ cells in the intestinal microbiome. Enrichment broths with supplementary antibiotics have been used elsewhere to aid detection of resistant organisms in faecal samples [18]. Our clinical experience in these hospitals suggests there are substantial challenges with over-crowding, limited access to hand-hygiene facilities and high patient:staff ratios. With regard to hand-hygiene, at the time the study was conducted, water supplies in these wards were sporadic and alcohol-based hand rub (ABHR) was not widely available. Concurrent work conducted in the same wards as a separate part of this pilot study showed that hands of adult carers were frequently contaminated with Gram-negative antibiotic resistant bacteria [19]. These are all factors that might be expected to contribute to exogenous routes of transmission.

In line with findings from other studies, we found that most ESBL-producing bacteria from these children were also resistant to several other clinically-relevant antibiotic classes [9–13]. This represents a substantial clinical challenge for empirical treatment of suspected hospital-acquired infections—ciprofloxacin (for severe urinary sepsis) and chloramphenicol (for bacteraemia and meningitis) represent important and widely used second-line treatment options in many African hospitals. The level of carbapenem resistance amongst resistant isolates was very low, but as these agents are not currently routinely available through public-sector pharmacies in Zimbabwe, this information is of limited practical value for local clinicians.

Due to rapid turnover of patients, we were only able to collect follow-up samples in a moderate number of patients; 73 patients had only a single sample collected. Whilst this does not affect estimates for the time of admission, it limits the ability of the study to detect new acquisition of carriage—in reality, probably even more children became carriers of resistance during admission than we detected. Ideally, an additional sample on the day of discharge would have been collected for all patients. Our simple screening and identification methods meant that we could not distinguish the simultaneous carriage of multiple different resistant organisms. Again, this means that the acquisition risk described here may underestimate the true in-hospital acquisition risk. We did not make use of molecular techniques for identification of resistance mechanisms or for investigating transmission of resistance. In this preliminary study, we were not able to evaluate clinical impacts of antibiotic resistance, either during hospital inpatient stay or after discharge. Whilst we did find some evidence that carriage of ESBL resistance was associated with inpatient mortality, investigating whether carriage of resistance was followed by clinical infection with the same organisms was beyond the scope of the study.

Future research is needed to better understand and tackle these high levels of antibiotic resistance. This will take many forms. We did not collect information on risk factors associated with carriage of resistance—this would be a valuable first step in understanding why this is occurring. In other settings, prior use of antibiotics and recent hospital admission have been associated with carriage of resistance [20]—we do not know if this is the case in Zimbabwe. More work is needed to identify the enzyme types and genes responsible for the forms of resistance that we detected—this will enable us to start to understand the dynamics of resistance transmission. Screening for drug-resistance on admission and cohorting or isolating patients can result in improved patient outcomes [21]—but no modelling work has ever been conducted to explore the value of this in the setting of African hospitals. We do not know how the acquisition of antibiotic resistance in hospitals relates to resistance in the community in Zimbabwe [22], nor do we know the wider impact of antibiotic resistance in terms of mortality, morbidity and costs in sub-Saharan African countries, though we suspect these to be substantial [23]. Given that many African hospitals have limited access to carbapenems, infections caused by ESBL-producers might have a substantially greater impact than has been seen in high-income settings [8].

At a more practical level, immediate action can be taken in a number of areas such as improving infection control practices, especially hand hygiene, which has been shown to reduce infections caused by antibiotic-resistant Gram-negative bacteria in high-income settings [24]. The use of alcohol-based hand rub as part of the WHO 5 moments for hand hygiene approach is a low-cost technology that could be much more widely adopted in African hospitals. Ideally, a rigorous evaluation of the implementation of such new practices would contribute to the evidence-base for these measures in low-income settings, which is currently extremely limited [25].

## Conclusion

The rising level of antimicrobial resistance is of great concern worldwide. These high rates of ESBL and gentamicin resistance carriage found amongst paediatric inpatients in Zimbabwe are particularly worrisome. These bacteria pose risks of multi-drug resistant infections and transmission to other hospital inpatients. The high levels of carriage at the time of hospital admissions suggest that they are already in widespread circulation in the general community. Relevant infection control measures such as access to alcohol-based hand rub should be urgently scaled up in these hospitals and beyond, so that children who arrive at hospital without carriage of antibiotic resistant bacteria can remain free of these for the duration of their admissions.

## Abbreviations

ABHR: Alcohol-based hand rub
CLSI: Clinical Laboratory Standards Institute
ESBL: Extended- spectrum β-lactamase
IQR: Inter-quartile range
